# The Cholera in New York

**Published:** 1887-10

**Authors:** 


### EDITORIAL DEPARTMENT.

F. E. DANIEL, M. D., EDITOR AND PUBLISHER

COLLABORATORS:

*E. J. Doering, M. D., Chicago.*
*Geo. Cupples, M. D., San Antonio.*
*Odo Betz, M. D., Germany.*
*E. J. Beall, M. D., Fort Worth, Texas.*
*T. C. Osborn, M. D., Cleburne, Texas.*
*Wm. Penny, M. D., New York.*
*H. O. Marcy, M. D., Boston.*
*C. K. Gregg, M. D., Mexico.*
*R. M. Swearingen. M. D., Austin.*
*C. H. Wilkinson, M. D., Galveston.*
*J. W. McLaughlin, M. D., Austin.*
*R. H. L. Bibb, M. D., Mexico.*

### THE CHOLERA IN NEW YORK.

When we reflect that it is a truth, a matter beyond dispute, that quarantine alone has never successfully barred the march of cholera, the landing of a cargo of five hundred passengers infected with the fearful plague, right into the heart of American population and commerce is enough to cause the stoutest heart to quake, and the bravest to tremble with fear! Think of it! Once passed the slim barrier opposed to it, and which has *so often* proved futile, perhaps one hundred thousand of our people will fall victims to its insatiate rage, and our fair land will become a charnel house and scene of deepest woe! Yet, strange apathy! the people seem as unconcerned as if cholera were at the moon!

We do not wish to be considered sensationists; but it were folly to shut our eyes to facts, and more than folly to have no realizing sense of the great and imminent danger now threatening us. We quote from an eminent English authority in the *British Medical Journal*, ("Prevention or Restriction of Cholera" by H. Thorne Thorne M. D., Lond.: F. R. C. S.)

"France is a quarantine country, and in 1884 she had full knowledge of the fact that her ports were in constant communication with Tonkin, where cholera was then prevailing. This, however, did not prevent the importation of the disease into Toulon in June of that year. Thence cholera spread to Marseilles and other

French cities and towns, until, by the close of the year, some five thousand deaths had taken place; this being followed by a renewed epidemic in 1885."

"Italy, learning of cholera in France, at once imposed measures of quarantine on all her coasts and frontiers. But the disease passed freely through the barrier, and entered by the sea—as well' as by land—first attacking the coast and frontier provinces. By the close of the year her official record told of 14,299 cholera deaths, 3,459 more following in 1885, the disease being maintained during 1886, and even to the present date.

"Algeria, only approachable from France by sea, imposed stringent measures of quarantine at all her ports, but the disease made its way in, by those very ports, and spread east and west along the coast line.

"Spain is a strictly quarantine country, and she, too, laid down rigid quarantine regulations against France, Italy, Algeria, and other countries. But cholera entered by a maritime province, the result being an initial epidemic in 1884, and no less than 119,620 deaths in 1885!

"So much for cholera prevention in countries resorting to their own approved measures of restriction."

These are fearful *facts;* and yet our health officers seem willing to try it again! to try to do what no other nation on earth has ever been able to do! As well might they endeavor to stop a locomotive with a brush broom! If they are doing *this*, and nothing more.

But, it will be asked—what else can we do? The first mistake was made, it is alleged, in permitting the Alesia to touch American soil. It is charged that the authorities should have turned the vessel back to sea with all those well persons on board, which would have been to condemn them to a certain fate. Such an act would have been in violation of the plainest dictates of common humanity. "Self preservation, 'they answer,' is the first law of life;" and they claim that in this civilized age we should do, as it is said the ancient Romans did, "sacrifice the few for the safety of the many.'

This is all wrong; and such doctrine is opposed to all reason; it would be as destructive to commerce, and more cruel than the shotgun quarantine. Such an act would shame a savage!

The landing of the vessel was all right, but the detention of the well was, and is, all wrong. They should have been permitted to go on their way, and doubtless by this time the disease would have been stamped out. The danger—the great danger, the great wrong and great injustice and inhumanity consist in *keeping those hundreds of well persons herded together*, fuel for the flame, almost sure to be consumed; and the longer they stay, the more infection will be generated, the more *foci* from which the disease will radiate, are created—and an epidemic will be almost surely ignited. The people of New York should rise up, and even now, demand their release; demand that all that kindling to the flame be scattered—that these people, so cruelly detained, should be permitted to go on their way rejoicing.

This may, to some, sound like a paradox, and to those who advocate non-intercourse, and who would send the Alesia adrift, it may seem absurd; this would apparently be the surest way to scatter the disease; but experience has demonstrated, time and again, that *inspection and isolation*, with *non*-detention of the well, is the only plan which has ever prevented the progress and spread of cholera. It is on the same principle that fire is limited by the removal of adjacent combustibles.

Let us to the proof, and to the plan dictated by reason, and approved by experience, in a country which claims to be in the van of sanitary progress, and whose claim is braced by statistics which must convince even the most skeptical.

England is the only country on earth that has, so far as we are aware, succeeded in keeping out the cholera without absolute non-intercourse.

The writer just quoted, says: "But what is our alternative system? Having deliberately abandoned quarantine, we began, years ago, to organize the system of medical inspection, with isolation. The medical inspection comes first into operation on our coast. The custom officers board the vessels coming into our ports, and they communicate to sanitary authority the occurrence of any case of cholera, choleraic diarrhœa, or suspected cholera. A vessel so affected is detained until the medical officer of health has examined every member of the crew and passengers. Those actually sick of cholera, or choleraic diarrhœa, are at once removed to the port sanitary hospital, and any person certified to be suffering from any illness which that officer suspects may prove to be cholera, is detained for a true observation, not exceeding two days. The medical inspection is thus followed by isolation of the sick. Unlike a quarantine system, this process does not interfere with the healthy, *or expose them to risk by herding them together with the sick.* [Italics ours. Here is the point, the danger, the mistake *if our men are making it.* Ed.] But the names of the healthy, and the place of their destination is taken, and the medical officers of health of the district in question are informed of the impending arrivals. This part of our system has been named the first line of defence; but it would be of little value if we stopped there. Our main trust is in the promotion of such local sanitary administration in every part of the country as shall rid us of the conditions under which alone cholera can spread. In periods of emergency, as during the past three years, a special medical survey of such districts as seem most exposed to risk is organized under the supervision of a medical officer of the local Government Board, and where needed the sanitary authorities are urged to action. Important as have been the results of the recent survey, they would go for little were it not for the steadily maintained work of sanitary authorities, and their officers throughout the kingdom; and we who have been taunted abroad for opposing quarantine because its restrictions touched our commercial interests and our pockets, may justly feel proud that in England and Wales alone the people have, during the past ten years, of their own accord, and apart from government dictation, spent, by way of loan, or in current expenditure, over eighty millions sterling for purposes mainly of a sanitary character."

The writer claims that that kingdom "still takes the lead as a progressive power in matters of public health;" and says: "the cost incurred has been immense, but it has not been in vain, or unremunerative." He then goes on to show that the cholera death rate in Great Britain, which was, in 1840, 30 per 10,000 inhabitants, has been reduced to 11 in 1854, and to 7 in 1886. "Since then," Dr. Thorne says "we have labored hard to prevent the disease from securing a footing amongst us; and though our labors are far from complete, and the disease has, *on several occasions, been imported, yet it has each time been at once checked.*" [Italics ours. Ed.]

We do not see how the sanitarians of this government, in knowledge of the facts above set forth, can hesitate to at once set in operation the plan, the only plan, which has been repeatedly demonstrated to be efficient in the limitation of cholera, if it be not already in operation—and to enforce the most rigid sanitation. It is absurd to say that the medical officer in charge, Dr. Smith, who is an advanced thinker, and recognized as a sanitarian of high order of ability, is a "dolt," as is so freely and so ignorantly charged by the sensation newspaper scribes. We believe that he has a keen appreciation both of the dangers, and the fearful responsibility resting on his head, and that whatever may be charged to the contrary, in ignorance of the facts, he is leaving nothing undone which is calculated to suppress the disease. We do not *know* what he is doing, but from his character, and well known ability and integrity, we assume that he is as competent to the task, and as honestly earnest in his efforts, and is putting them forth with at least as much intelligence as the average newspaper man, who is so ready to abuse him, would be capable of doing. But should it prove that such is not the case; that Dr. Smith is blindly or wilfully shutting his eyes to the lessons taught by the failure of quarantine in other countries, and the success of the British plan, and an epidemic should be the result, the responsibility should be severely visited upon his head.

It will not do to trust to quarantine alone; for it is demonstrably true that quarantining countries are essentially those which cholera invades.

If further proof were needed, that well persons will not scatter the disease, if proper precautions are taken, we would cite the outbreak of cholera at Niagara City in '75, reported by the late Dr. Frank H. Hamilton in *New York Medical Journal*. A body of German emigrants just landed, camped on the banks of the stream. That night one of the party died, and the village being in an unsanitary condition, an epidemic lighted up which nearly depopulated the place; but the well persons of the party took the train next day and proceeded west, and not another case occurred.

